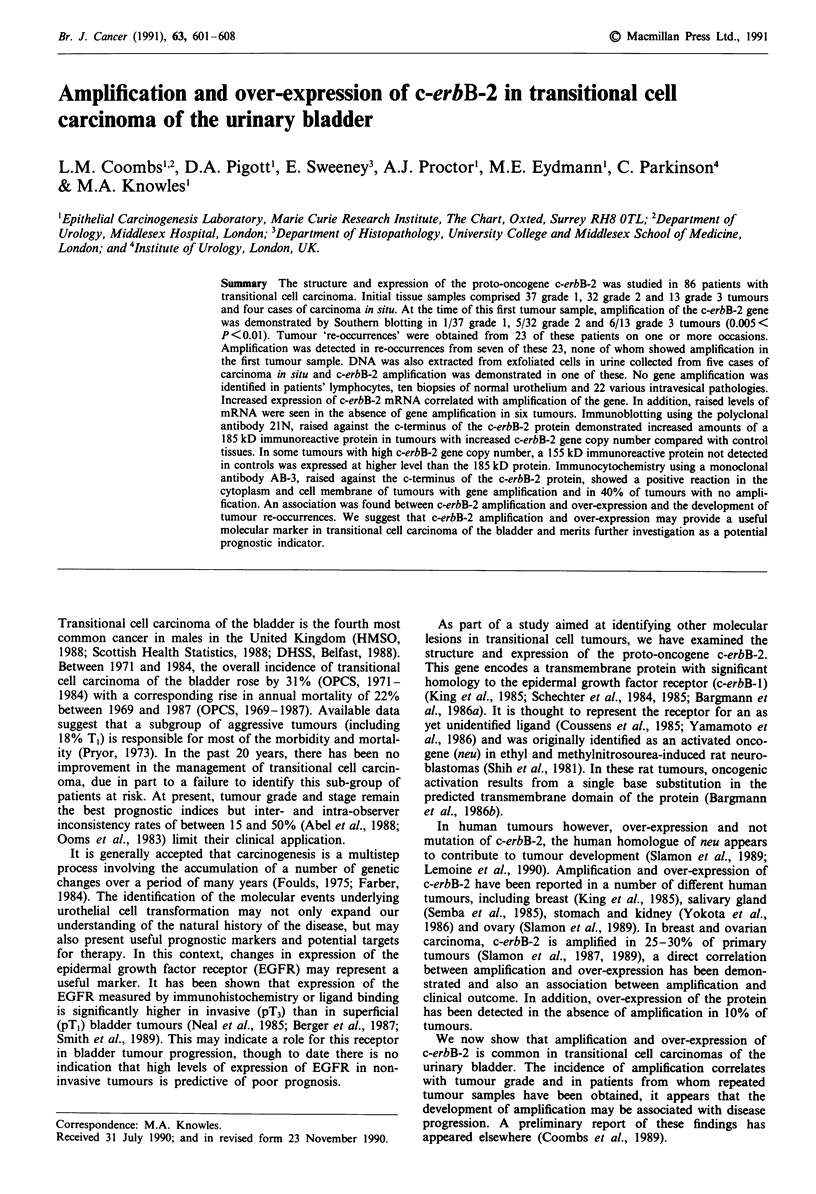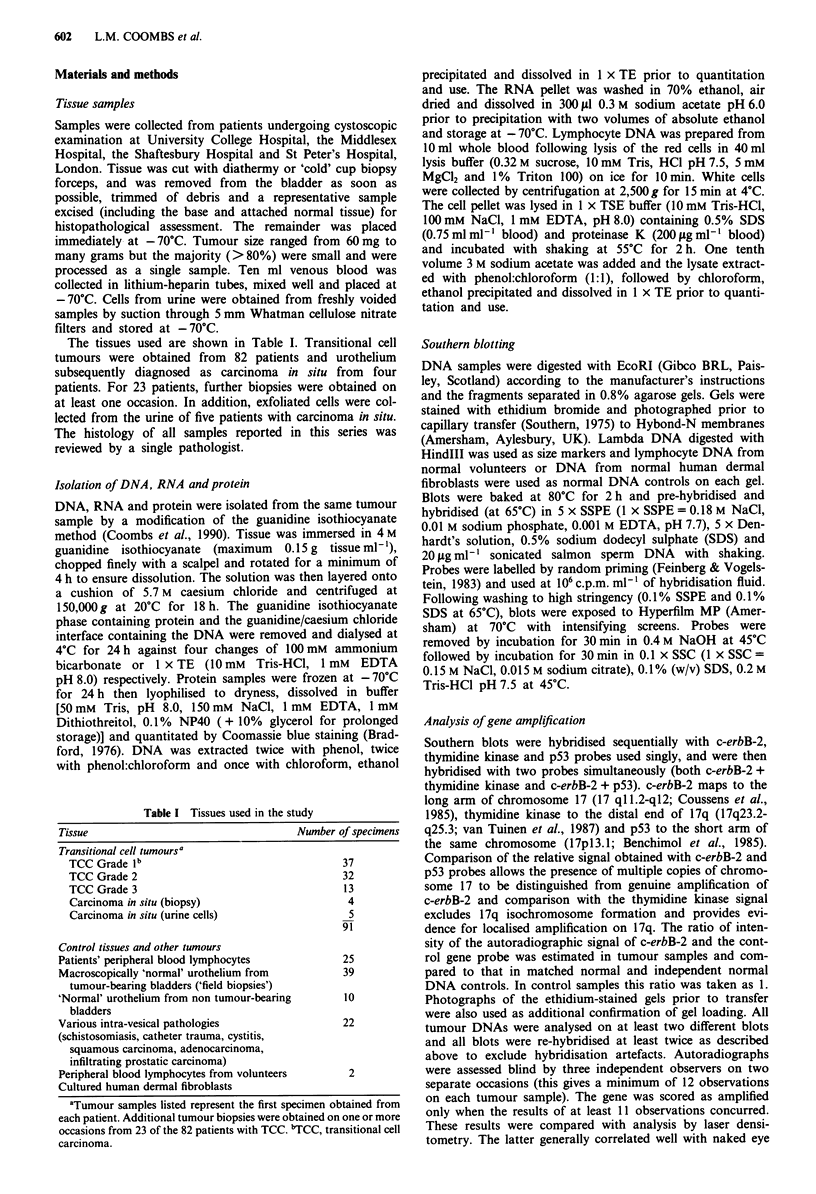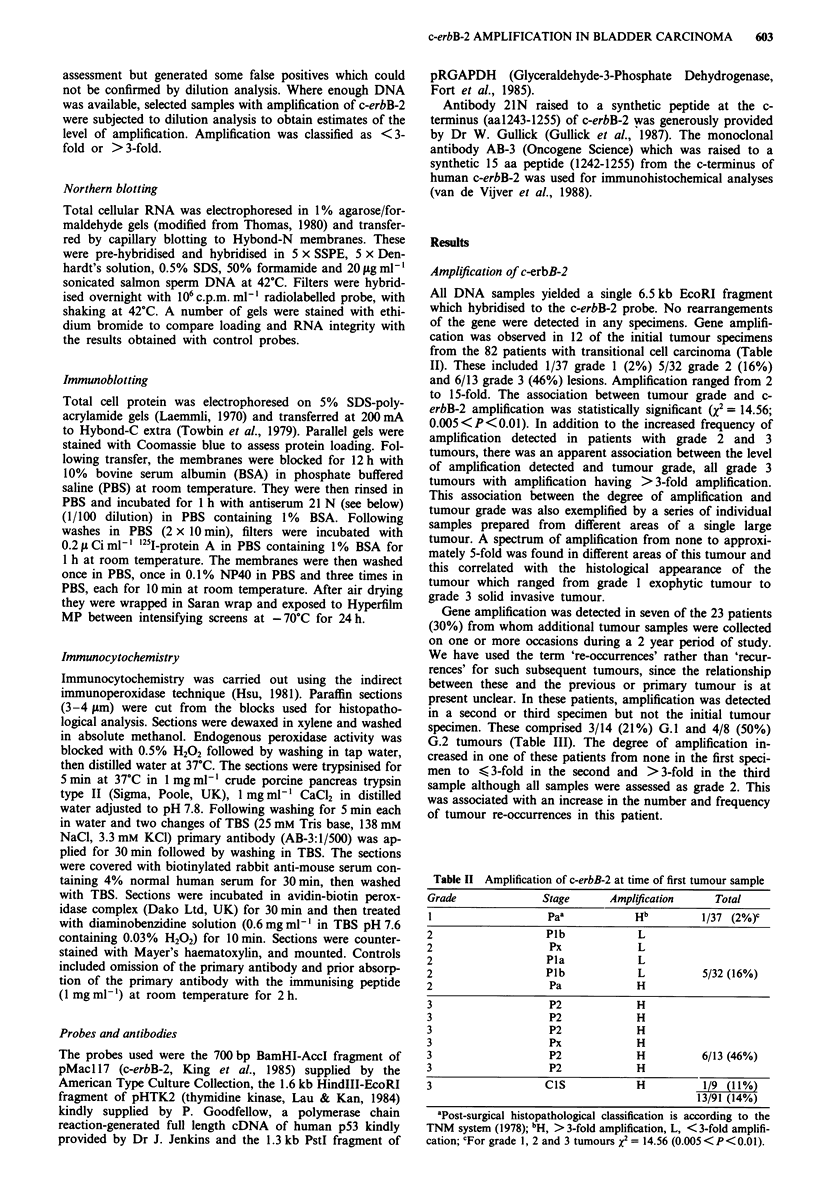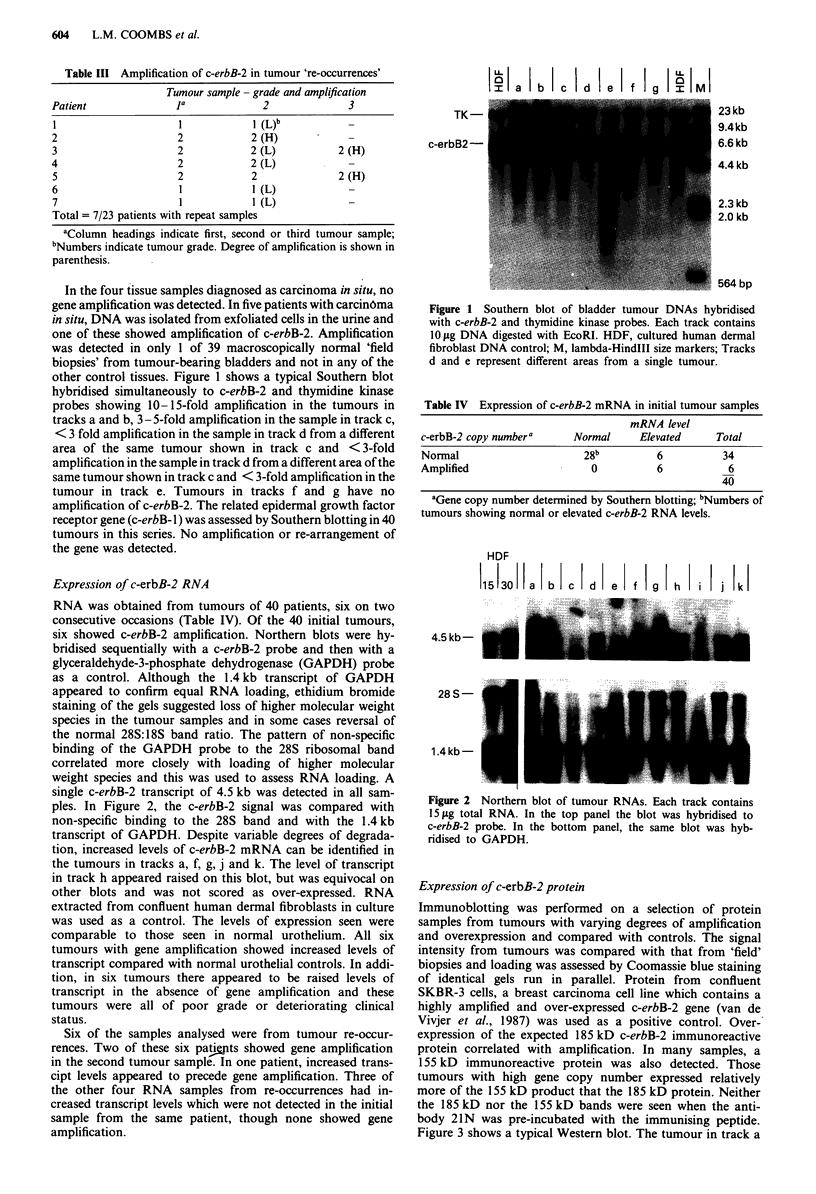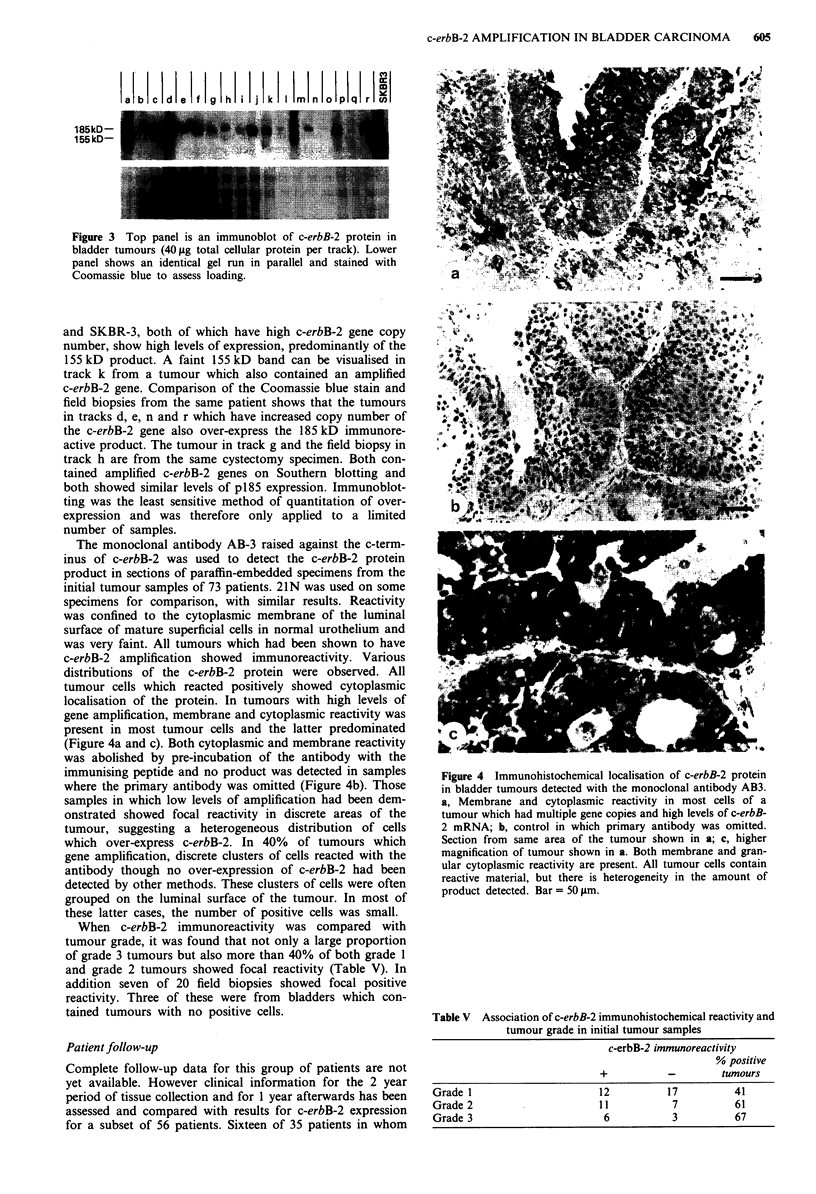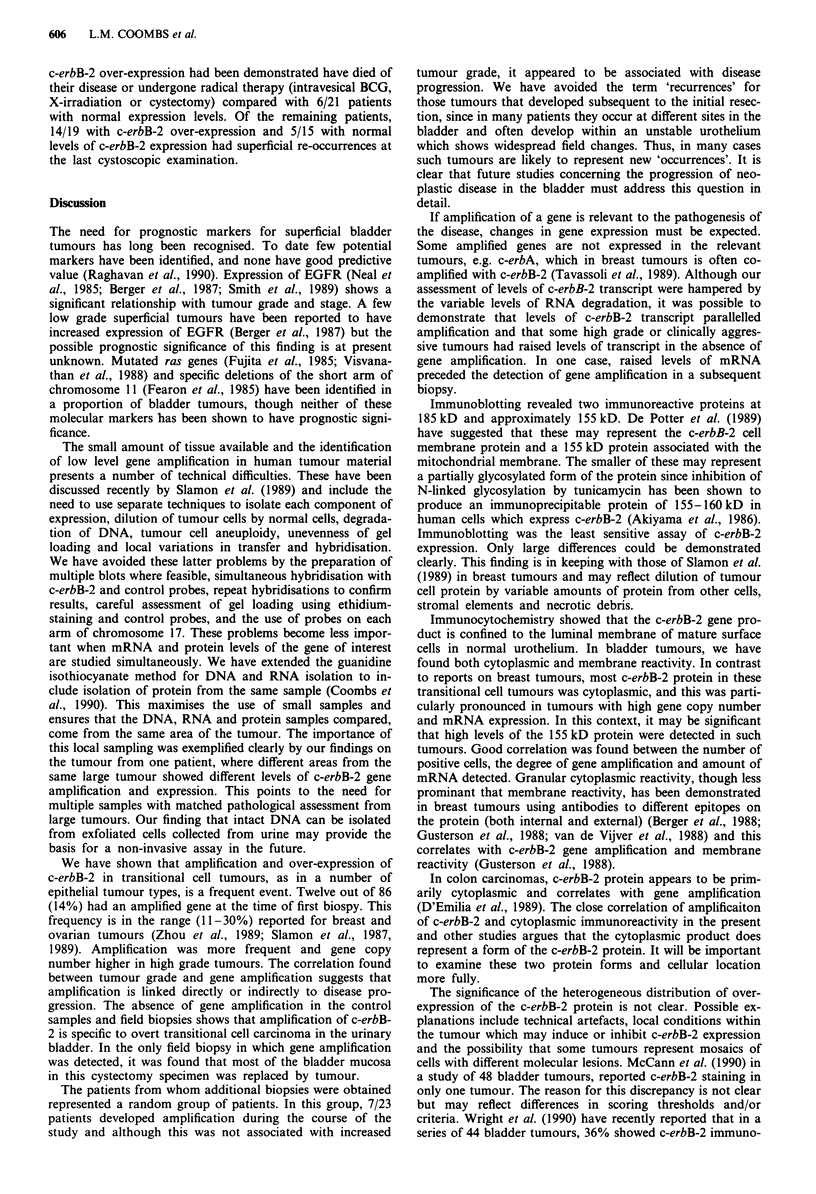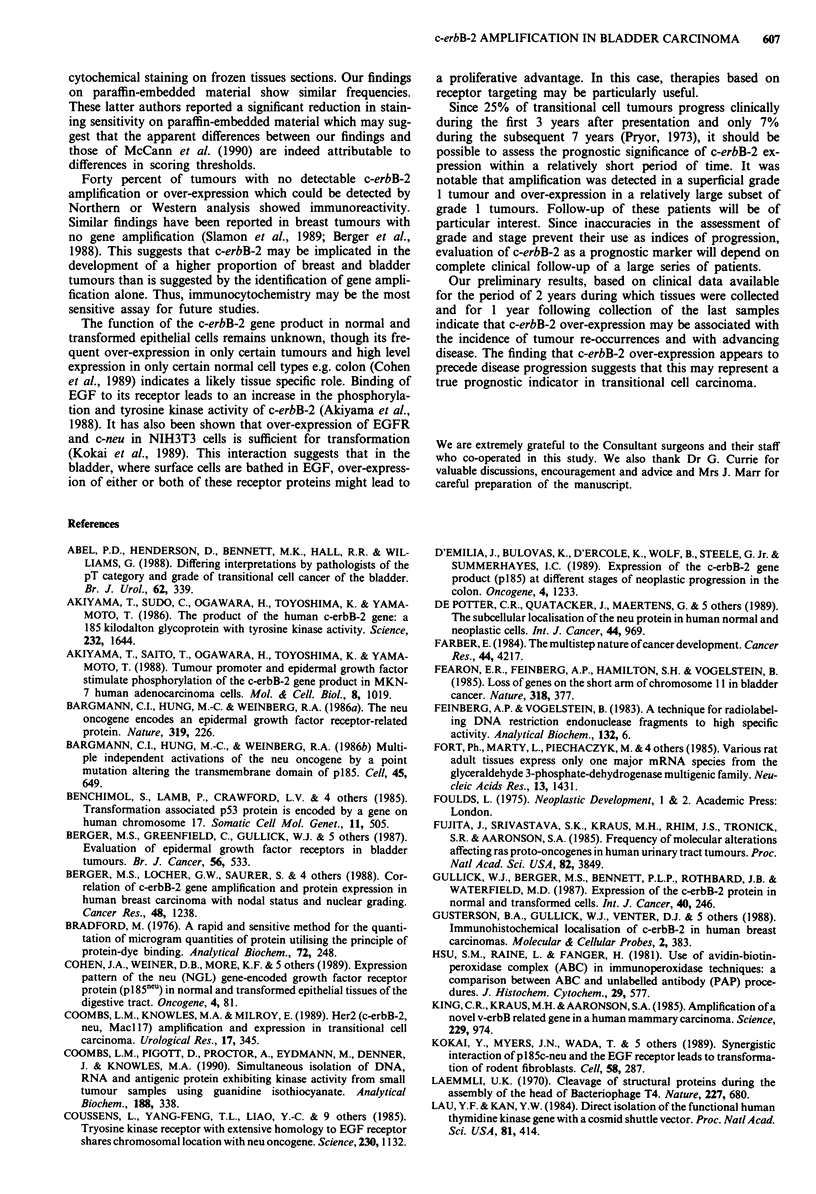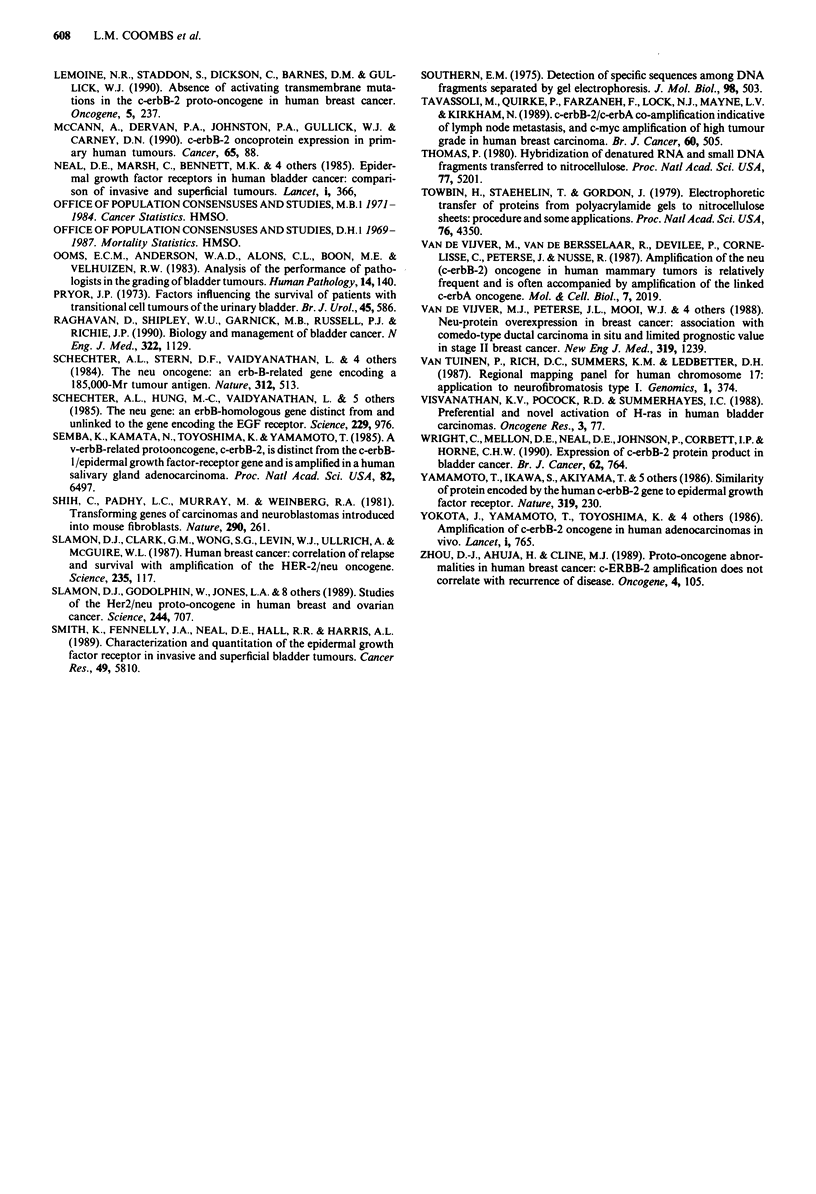# Amplification and over-expression of c-erbB-2 in transitional cell carcinoma of the urinary bladder.

**DOI:** 10.1038/bjc.1991.139

**Published:** 1991-04

**Authors:** L. M. Coombs, D. A. Pigott, E. Sweeney, A. J. Proctor, M. E. Eydmann, C. Parkinson, M. A. Knowles

**Affiliations:** Epithelial Carcinogenesis Laboratory, Marie Curie Research Institute, Oxted, Surrey, UK.

## Abstract

**Images:**


Br. J. Cancer (1991), 63, 601–608 © Macmillan Press Ltd., 1991

### Amplification and over-expression of c-*erb*B-2 in transitional cell carcinoma of the urinary bladder

L.M. Coombs[1,2], D.A. Pigott[1], E. Sweeney[3], A.J. Proctor[1], M.E. Eydmann[1], C. Parkinson[4] & M.A. Knowles[1]

[1]*Epithelial Carcinogenesis Laboratory, Marie Curie Research Institute, The Chart, Oxted, Surrey RH8 0TL;* [2]*Department of Urology, Middlesex Hospital, London;* [3]*Department of Histopathology, University College and Middlesex School of Medicine, London; and* [4]*Institute of Urology, London, UK.*

**Summary** The structure and expression of the proto-oncogene c-*erb*B-2 was studied in 86 patients with transitional cell carcinoma. Initial tissue samples comprised 37 grade 1, 32 grade 2 and 13 grade 3 tumours and four cases of carcinoma *in situ*. At the time of this first tumour sample, amplification of the c-*erb*B-2 gene was demonstrated by Southern blotting in 1/37 grade 1, 5/32 grade 2 and 6/13 grade 3 tumours ($0.005 < P < 0.01$). Tumour 're-occurrences' were obtained from 23 of these patients on one or more occasions. Amplification was detected in re-occurrences from seven of these 23, none of whom showed amplification in the first tumour sample. DNA was also extracted from exfoliated cells in urine collected from five cases of carcinoma *in situ* and c-*erb*B-2 amplification was demonstrated in one of these. No gene amplification was identified in patients' lymphocytes, ten biopsies of normal urothelium and 22 various intravesical pathologies. Increased expression of c-*erb*B-2 mRNA correlated with amplification of the gene. In addition, raised levels of mRNA were seen in the absence of gene amplification in six tumours. Immunoblotting using the polyclonal antibody 21N, raised against the c-terminus of the c-*erb*B-2 protein demonstrated increased amounts of a 185 kD immunoreactive protein in tumours with increased c-*erb*B-2 gene copy number compared with control tissues. In some tumours with high c-*erb*B-2 gene copy number, a 155 kD immunoreactive protein not detected in controls was expressed at higher level than the 185 kD protein. Immunocytochemistry using a monoclonal antibody AB-3, raised against the c-terminus of the c-*erb*B-2 protein, showed a positive reaction in the cytoplasm and cell membrane of tumours with gene amplification and in 40% of tumours with no amplification. An association was found between c-*erb*B-2 amplification and over-expression and the development of tumour re-occurrences. We suggest that c-*erb*B-2 amplification and over-expression may provide a useful molecular marker in transitional cell carcinoma of the bladder and merits further investigation as a potential prognostic indicator.

Transitional cell carcinoma of the bladder is the fourth most common cancer in males in the United Kingdom (HMSO, 1988; Scottish Health Statistics, 1988; DHSS, Belfast, 1988). Between 1971 and 1984, the overall incidence of transitional cell carcinoma of the bladder rose by 31% (OPCS, 1971–1984) with a corresponding rise in annual mortality of 22% between 1969 and 1987 (OPCS, 1969–1987). Available data suggest that a subgroup of aggressive tumours (including 18% $T_1$) is responsible for most of the morbidity and mortality (Pryor, 1973). In the past 20 years, there has been no improvement in the management of transitional cell carcinoma, due in part to a failure to identify this sub-group of patients at risk. At present, tumour grade and stage remain the best prognostic indices but inter- and intra-observer inconsistency rates of between 15 and 50% (Abel *et al.*, 1988; Ooms *et al.*, 1983) limit their clinical application.

It is generally accepted that carcinogenesis is a multistep process involving the accumulation of a number of genetic changes over a period of many years (Foulds, 1975; Farber, 1984). The identification of the molecular events underlying urothelial cell transformation may not only expand our understanding of the natural history of the disease, but may also present useful prognostic markers and potential targets for therapy. In this context, changes in expression of the epidermal growth factor receptor (EGFR) may represent a useful marker. It has been shown that expression of the EGFR measured by immunohistochemistry or ligand binding is significantly higher in invasive ($pT_3$) than in superficial ($pT_1$) bladder tumours (Neal *et al.*, 1985; Berger *et al.*, 1987; Smith *et al.*, 1989). This may indicate a role for this receptor in bladder tumour progression, though to date there is no indication that high levels of expression of EGFR in non-invasive tumours is predictive of poor prognosis.

Correspondence: M.A. Knowles.
Received 31 July 1990; and in revised form 23 November 1990.

As part of a study aimed at identifying other molecular lesions in transitional cell tumours, we have examined the structure and expression of the proto-oncogene c-*erb*B-2. This gene encodes a transmembrane protein with significant homology to the epidermal growth factor receptor (c-*erb*B-1) (King *et al.*, 1985; Schechter *et al.*, 1984, 1985; Bargmann *et al.*, 1986*a*). It is thought to represent the receptor for an as yet unidentified ligand (Coussens *et al.*, 1985; Yamamoto *et al.*, 1986) and was originally identified as an activated oncogene (*neu*) in ethyl and methylnitrosourea-induced rat neuroblastomas (Shih *et al.*, 1981). In these rat tumours, oncogenic activation results from a single base substitution in the predicted transmembrane domain of the protein (Bargmann *et al.*, 1986*b*).

In human tumours however, over-expression and not mutation of c-*erb*B-2, the human homologue of *neu* appears to contribute to tumour development (Slamon *et al.*, 1989; Lemoine *et al.*, 1990). Amplification and over-expression of c-*erb*B-2 have been reported in a number of different human tumours, including breast (King *et al.*, 1985), salivary gland (Semba *et al.*, 1985), stomach and kidney (Yokota *et al.*, 1986) and ovary (Slamon *et al.*, 1989). In breast and ovarian carcinoma, c-*erb*B-2 is amplified in 25–30% of primary tumours (Slamon *et al.*, 1987, 1989), a direct correlation between amplification and over-expression has been demonstrated and also an association between amplification and clinical outcome. In addition, over-expression of the protein has been detected in the absence of amplification in 10% of tumours.

We now show that amplification and over-expression of c-*erb*B-2 is common in transitional cell carcinomas of the urinary bladder. The incidence of amplification correlates with tumour grade and in patients from whom repeated tumour samples have been obtained, it appears that the development of amplification may be associated with disease progression. A preliminary report of these findings has appeared elsewhere (Coombs *et al.*, 1989).

### Materials and methods

### Tissue samples

Samples were collected from patients undergoing cystoscopic examination at University College Hospital, the Middlesex Hospital, the Shaftesbury Hospital and St Peter's Hospital, London. Tissue was cut with diathermy or 'cold' cup biopsy forceps, and was removed from the bladder as soon as possible, trimmed of debris and a representative sample excised (including the base and attached normal tissue) for histopathological assessment. The remainder was placed immediately at $-70°C$. Tumour size ranged from 60 mg to many grams but the majority ($>80\%$) were small and were processed as a single sample. Ten ml venous blood was collected in lithium-heparin tubes, mixed well and placed at $-70°C$. Cells from urine were obtained from freshly voided samples by suction through 5 mm Whatman cellulose nitrate filters and stored at $-70°C$.

The tissues used are shown in Table I. Transitional cell tumours were obtained from 82 patients and urothelium subsequently diagnosed as carcinoma *in situ* from four patients. For 23 patients, further biopsies were obtained on at least one occasion. In addition, exfoliated cells were collected from the urine of five patients with carcinoma *in situ*. The histology of all samples reported in this series was reviewed by a single pathologist.

### Isolation of DNA, RNA and protein

DNA, RNA and protein were isolated from the same tumour sample by a modification of the guanidine isothiocyanate method (Coombs *et al.*, 1990). Tissue was immersed in 4 M guanidine isothiocyanate (maximum 0.15 g tissue $ml^{-1}$), chopped finely with a scalpel and rotated for a minimum of 4 h to ensure dissolution. The solution was then layered onto a cushion of 5.7 M caesium chloride and centrifuged at 150,000 *g* at 20°C for 18 h. The guanidine isothiocyanate phase containing protein and the guanidine/caesium chloride interface containing the DNA were removed and dialysed at 4°C for 24 h against four changes of 100 mM ammonium bicarbonate or 1 × TE (10 mM Tris-HCl, 1 mM EDTA pH 8.0) respectively. Protein samples were frozen at $-70°C$ for 24 h then lyophilised to dryness, dissolved in buffer [50 mM Tris, pH 8.0, 150 mM NaCl, 1 mM EDTA, 1 mM Dithiothreitol, 0.1% NP40 (+10% glycerol for prolonged storage)] and quantitated by Coomassie blue staining (Bradford, 1976). DNA was extracted twice with phenol, twice with phenol:chloroform and once with chloroform, ethanol precipitated and dissolved in 1 × TE prior to quantitation and use. The RNA pellet was washed in 70% ethanol, air dried and dissolved in 300 µl 0.3 M sodium acetate pH 6.0 prior to precipitation with two volumes of absolute ethanol and storage at $-70°C$. Lymphocyte DNA was prepared from 10 ml whole blood following lysis of the red cells in 40 ml lysis buffer (0.32 M sucrose, 10 mM Tris, HCl pH 7.5, 5 mM $MgCl_2$ and 1% Triton 100) on ice for 10 min. White cells were collected by centrifugation at 2,500 *g* for 15 min at 4°C. The cell pellet was lysed in 1 × TSE buffer (10 mM Tris-HCl, 100 mM NaCl, 1 mM EDTA, pH 8.0) containing 0.5% SDS (0.75 ml $ml^{-1}$ blood) and proteinase K (200 µg $ml^{-1}$ blood) and incubated with shaking at 55°C for 2 h. One tenth volume 3 M sodium acetate was added and the lysate extracted with phenol:chloroform (1:1), followed by chloroform, ethanol precipitated and dissolved in 1 × TE prior to quantitation and use.

**Table I** Tissues used in the study

| *Tissue* | *Number of specimens* |
|---|---|
| *Transitional cell tumours*[a] | |
| TCC Grade 1[b] | 37 |
| TCC Grade 2 | 32 |
| TCC Grade 3 | 13 |
| Carcinoma *in situ* (biopsy) | 4 |
| Carcinoma *in situ* (urine cells) | 5 |
| | 91 |
| *Control tissues and other tumours* | |
| Patients' peripheral blood lymphocytes | 25 |
| Macroscopically 'normal' urothelium from tumour-bearing bladders ('field biopsies') | 39 |
| 'Normal' urothelium from non tumour-bearing bladders | 10 |
| Various intra-vesical pathologies (schistosomiasis, catheter trauma, cystitis, squamous carcinoma, adenocarcinoma, infiltrating prostatic carcinoma) | 22 |
| Peripheral blood lymphocytes from volunteers | 2 |
| Cultured human dermal fibroblasts | |

[a]Tumour samples listed represent the first specimen obtained from each patient. Additional tumour biopsies were obtained on one or more occasions from 23 of the 82 patients with TCC. [b]TCC, transitional cell carcinoma.

### Southern blotting

DNA samples were digested with EcoRI (Gibco BRL, Paisley, Scotland) according to the manufacturer's instructions and the fragments separated in 0.8% agarose gels. Gels were stained with ethidium bromide and photographed prior to capillary transfer (Southern, 1975) to Hybond-N membranes (Amersham, Aylesbury, UK). Lambda DNA digested with HindIII was used as size markers and lymphocyte DNA from normal volunteers or DNA from normal human dermal fibroblasts were used as normal DNA controls on each gel. Blots were baked at 80°C for 2 h and pre-hybridised and hybridised (at 65°C) in 5 × SSPE (1 × SSPE = 0.18 M NaCl, 0.01 M sodium phosphate, 0.001 M EDTA, pH 7.7), 5 × Denhardt's solution, 0.5% sodium dodecyl sulphate (SDS) and 20 µg $ml^{-1}$ sonicated salmon sperm DNA with shaking. Probes were labelled by random priming (Feinberg & Vogelstein, 1983) and used at $10^6$ c.p.m. $ml^{-1}$ of hybridisation fluid. Following washing to high stringency (0.1% SSPE and 0.1% SDS at 65°C), blots were exposed to Hyperfilm MP (Amersham) at 70°C with intensifying screens. Probes were removed by incubation for 30 min in 0.4 M NaOH at 45°C followed by incubation for 30 min in 0.1 × SSC (1 × SSC = 0.15 M NaCl, 0.015 M sodium citrate), 0.1% (w/v) SDS, 0.2 M Tris-HCl pH 7.5 at 45°C.

### Analysis of gene amplification

Southern blots were hybridised sequentially with c-*erb*B-2, thymidine kinase and p53 probes used singly, and were then hybridised with two probes simultaneously (both c-*erb*B-2 + thymidine kinase and c-*erb*B-2 + p53). c-*erb*B-2 maps to the long arm of chromosome 17 (17 q11.2-q12; Coussens *et al.*, 1985), thymidine kinase to the distal end of 17q (17q23.2-q25.3; van Tuinen *et al.*, 1987) and p53 to the short arm of the same chromosome (17p13.1; Benchimol *et al.*, 1985). Comparison of the relative signal obtained with c-*erb*B-2 and p53 probes allows the presence of multiple copies of chromosome 17 to be distinguished from genuine amplification of c-*erb*B-2 and comparison with the thymidine kinase signal excludes 17q isochromosome formation and provides evidence for localised amplification on 17q. The ratio of intensity of the autoradiographic signal of c-*erb*B-2 and the control gene probe was estimated in tumour samples and compared to that in matched normal and independent normal DNA controls. In control samples this ratio was taken as 1. Photographs of the ethidium-stained gels prior to transfer were also used as additional confirmation of gel loading. All tumour DNAs were analysed on at least two different blots and all blots were re-hybridised at least twice as described above to exclude hybridisation artefacts. Autoradiographs were assessed blind by three independent observers on two separate occasions (this gives a minimum of 12 observations on each tumour sample). The gene was scored as amplified only when the results of at least 11 observations concurred. These results were compared with analysis by laser densitometry. The latter generally correlated well with naked eye assessment but generated some false positives which could not be confirmed by dilution analysis. Where enough DNA was available, selected samples with amplification of c-*erb*B-2 were subjected to dilution analysis to obtain estimates of the level of amplification. Amplification was classified as <3-fold or >3-fold.

*Northern blotting*

Total cellular RNA was electrophoresed in 1% agarose/formaldehyde gels (modified from Thomas, 1980) and transferred by capillary blotting to Hybond-N membranes. These were pre-hybridised and hybridised in 5 x SSPE, 5 x Denhardt's solution, 0.5% SDS, 50% formamide and 20 $\mu g\ ml^{-1}$ sonicated salmon sperm DNA at 42°C. Filters were hybridised overnight with $10^6$ c.p.m. $ml^{-1}$ radiolabelled probe, with shaking at 42°C. A number of gels were stained with ethidium bromide to compare loading and RNA integrity with the results obtained with control probes.

*Immunoblotting*

Total cell protein was electrophoresed on 5% SDS-polyacrylamide gels (Laemmli, 1970) and transferred at 200 mA to Hybond-C extra (Towbin *et al*., 1979). Parallel gels were stained with Coomassie blue to assess protein loading. Following transfer, the membranes were blocked for 12 h with 10% bovine serum albumin (BSA) in phosphate buffered saline (PBS) at room temperature. They were then rinsed in PBS and incubated for 1 h with antiserum 21 N (see below) (1/100 dilution) in PBS containing 1% BSA. Following washes in PBS (2 x 10 min), filters were incubated with 0.2 $\mu Ci\ ml^{-1}$ $^{125}I$-protein A in PBS containing 1% BSA for 1 h at room temperature. The membranes were then washed once in PBS, once in 0.1% NP40 in PBS and three times in PBS, each for 10 min at room temperature. After air drying they were wrapped in Saran wrap and exposed to Hyperfilm MP between intensifying screens at −70°C for 24 h.

*Immunocytochemistry*

Immunocytochemistry was carried out using the indirect immunoperoxidase technique (Hsu, 1981). Paraffin sections (3–4 μm) were cut from the blocks used for histopathological analysis. Sections were dewaxed in xylene and washed in absolute methanol. Endogenous peroxidase activity was blocked with 0.5% $H_2O_2$ followed by washing in tap water, then distilled water at 37°C. The sections were trypsinised for 5 min at 37°C in 1 mg $ml^{-1}$ crude porcine pancreas trypsin type II (Sigma, Poole, UK), 1 mg $ml^{-1}$ $CaCl_2$ in distilled water adjusted to pH 7.8. Following washing for 5 min each in water and two changes of TBS (25 mM Tris base, 138 mM NaCl, 3.3 mM KCl) primary antibody (AB-3:1/500) was applied for 30 min followed by washing in TBS. The sections were covered with biotinylated rabbit anti-mouse serum containing 4% normal human serum for 30 min, then washed with TBS. Sections were incubated in avidin-biotin peroxidase complex (Dako Ltd, UK) for 30 min and then treated with diaminobenzidine solution (0.6 mg $ml^{-1}$ in TBS pH 7.6 containing 0.03% $H_2O_2$) for 10 min. Sections were counterstained with Mayer's haematoxylin, and mounted. Controls included omission of the primary antibody and prior absorption of the primary antibody with the immunising peptide (1 mg $ml^{-1}$) at room temperature for 2 h.

*Probes and antibodies*

The probes used were the 700 bp BamHI-AccI fragment of pMac117 (c-*erb*B-2, King *et al*., 1985) supplied by the American Type Culture Collection, the 1.6 kb HindIII-EcoRI fragment of pHTK2 (thymidine kinase, Lau & Kan, 1984) kindly supplied by P. Goodfellow, a polymerase chain reaction-generated full length cDNA of human p53 kindly provided by Dr J. Jenkins and the 1.3 kb PstI fragment of pRGAPDH (Glyceraldehyde-3-Phosphate Dehydrogenase, Fort *et al*., 1985).

Antibody 21N raised to a synthetic peptide at the c-terminus (aa1243-1255) of c-*erb*B-2 was generously provided by Dr W. Gullick (Gullick *et al*., 1987). The monoclonal antibody AB-3 (Oncogene Science) which was raised to a synthetic 15 aa peptide (1242-1255) from the c-terminus of human c-*erb*B-2 was used for immunohistochemical analyses (van de Vijver *et al*., 1988).

### Results

*Amplification of c-erbB-2*

All DNA samples yielded a single 6.5 kb EcoRI fragment which hybridised to the c-*erb*B-2 probe. No rearrangements of the gene were detected in any specimens. Gene amplification was observed in 12 of the initial tumour specimens from the 82 patients with transitional cell carcinoma (Table II). These included 1/37 grade 1 (2%) 5/32 grade 2 (16%) and 6/13 grade 3 (46%) lesions. Amplification ranged from 2 to 15-fold. The association between tumour grade and c-*erb*B-2 amplification was statistically significant ($\chi^2 = 14.56$; $0.005 < P < 0.01$). In addition to the increased frequency of amplification detected in patients with grade 2 and 3 tumours, there was an apparent association between the level of amplification detected and tumour grade, all grade 3 tumours with amplification having >3-fold amplification. This association between the degree of amplification and tumour grade was also exemplified by a series of individual samples prepared from different areas of a single large tumour. A spectrum of amplification from none to approximately 5-fold was found in different areas of this tumour and this correlated with the histological appearance of the tumour which ranged from grade 1 exophytic tumour to grade 3 solid invasive tumour.

Gene amplification was detected in seven of the 23 patients (30%) from whom additional tumour samples were collected on one or more occasions during a 2 year period of study. We have used the term 're-occurrences' rather than 'recurrences' for such subsequent tumours, since the relationship between these and the previous or primary tumour is at present unclear. In these patients, amplification was detected in a second or third specimen but not the initial tumour specimen. These comprised 3/14 (21%) G.1 and 4/8 (50%) G.2 tumours (Table III). The degree of amplification increased in one of these patients from none in the first specimen to ⩽3-fold in the second and >3-fold in the third sample although all samples were assessed as grade 2. This was associated with an increase in the number and frequency of tumour re-occurrences in this patient.

**Table II** Amplification of c-*erbB*-2 at time of first tumour sample

| *Grade* | *Stage* | *Amplification* | *Total* |
|---|---|---|---|
| 1 | Pa[a] | H[b] | 1/37 (2%)[c] |
| 2 | P1b | L | |
| 2 | Px | L | |
| 2 | P1a | L | |
| 2 | P1b | L | 5/32 (16%) |
| 2 | Pa | H | |
| 3 | P2 | H | |
| 3 | P2 | H | |
| 3 | P2 | H | |
| 3 | Px | H | |
| 3 | P2 | H | 6/13 (46%) |
| 3 | P2 | H | |
| 3 | CIS | H | 1/9 (11%) |
| | | | 13/91 (14%) |

[a]Post-surgical histopathological classification is according to the TNM system (1978); [b]H, >3-fold amplification, L, <3-fold amplification; [c]For grade 1, 2 and 3 tumours $\chi^2 = 14.56$ ($0.005 < P < 0.01$).

**Table III** Amplification of c-*erbB*-2 in tumour 're-occurrences'

| Patient | *Tumour sample – grade and amplification* 1[a] | 2 | 3 |
|---|---|---|---|
| 1 | 1 | 1 (L)[b] | – |
| 2 | 2 | 2 (H) | – |
| 3 | 2 | 2 (L) | 2 (H) |
| 4 | 2 | 2 (L) | – |
| 5 | 2 | 2 | 2 (H) |
| 6 | 1 | 1 (L) | – |
| 7 | 1 | 1 (L) | – |
| Total = 7/23 patients with repeat samples | | | |

[a]Column headings indicate first, second or third tumour sample; [b]Numbers indicate tumour grade. Degree of amplification is shown in parenthesis.

In the four tissue samples diagnosed as carcinoma *in situ*, no gene amplification was detected. In five patients with carcinoma *in situ*, DNA was isolated from exfoliated cells in the urine and one of these showed amplification of c-*erb*B-2. Amplification was detected in only 1 of 39 macroscopically normal 'field biopsies' from tumour-bearing bladders and not in any of the other control tissues. Figure 1 shows a typical Southern blot hybridised simultaneously to c-*erb*B-2 and thymidine kinase probes showing 10–15-fold amplification in the tumours in tracks a and b, 3–5-fold amplification in the sample in track c, $<3$ fold amplification in the sample in track d from a different area of the same tumour shown in track c and $<3$-fold amplification in the sample in track d from a different area of the same tumour shown in track c and $<3$-fold amplification in the tumour in track e. Tumours in tracks f and g have no amplification of c-*erb*B-2. The related epidermal growth factor receptor gene (c-*erb*B-1) was assessed by Southern blotting in 40 tumours in this series. No amplification or re-arrangement of the gene was detected.

*Expression of* c-erb*B-2 RNA*

RNA was obtained from tumours of 40 patients, six on two consecutive occasions (Table IV). Of the 40 initial tumours, six showed c-*erb*B-2 amplification. Northern blots were hybridised sequentially with a c-*erb*B-2 probe and then with a glyceraldehyde-3-phosphate dehydrogenase (GAPDH) probe as a control. Although the 1.4 kb transcript of GAPDH appeared to confirm equal RNA loading, ethidium bromide staining of the gels suggested loss of higher molecular weight species in the tumour samples and in some cases reversal of the normal 28S:18S band ratio. The pattern of non-specific binding of the GAPDH probe to the 28S ribosomal band correlated more closely with loading of higher molecular weight species and this was used to assess RNA loading. A single c-*erb*B-2 transcript of 4.5 kb was detected in all samples. In Figure 2, the c-*erb*B-2 signal was compared with non-specific binding to the 28S band and with the 1.4 kb transcript of GAPDH. Despite variable degrees of degradation, increased levels of c-*erb*B-2 mRNA can be identified in the tumours in tracks a, f, g, j and k. The level of transcript in track h appeared raised on this blot, but was equivocal on other blots and was not scored as over-expressed. RNA extracted from confluent human dermal fibroblasts in culture was used as a control. The levels of expression seen were comparable to those seen in normal urothelium. All six tumours with gene amplification showed increased levels of transcript compared with normal urothelial controls. In addition, in six tumours there appeared to be raised levels of transcript in the absence of gene amplification and these tumours were all of poor grade or deteriorating clinical status.

Six of the samples analysed were from tumour re-occurrences. Two of these six patients showed gene amplification in the second tumour sample. In one patient, increased transcipt levels appeared to precede gene amplification. Three of the other four RNA samples from re-occurrences had increased transcript levels which were not detected in the initial sample from the same patient, though none showed gene amplification.

**Figure 1** Southern blot of bladder tumour DNAs hybridised with c-*erbB*-2 and thymidine kinase probes. Each track contains 10 μg DNA digested with EcoRI. HDF, cultured human dermal fibroblast DNA control; M, lambda-HindIII size markers; Tracks d and e represent different areas from a single tumour.

**Table IV** Expression of c-*erbB*-2 mRNA in initial tumour samples

| c-erbB-*2 copy number*[a] | *mRNA level* *Normal* | *Elevated* | *Total* |
|---|---|---|---|
| Normal | 28[b] | 6 | 34 |
| Amplified | 0 | 6 | 6 |
| | | | 40 |

[a]Gene copy number determined by Southern blotting; [b]Numbers of tumours showing normal or elevated c-*erbB*-2 RNA levels.

**Figure 2** Northern blot of tumour RNAs. Each track contains 15 μg total RNA. In the top panel the blot was hybridised to c-*erbB*-2 probe. In the bottom panel, the same blot was hybridised to GAPDH.

*Expression of* c-erb*B-2 protein*

Immunoblotting was performed on a selection of protein samples from tumours with varying degrees of amplification and overexpression and compared with controls. The signal intensity from tumours was compared with that from 'field' biopsies and loading was assessed by Coomassie blue staining of identical gels run in parallel. Protein from confluent SKBR-3 cells, a breast carcinoma cell line which contains a highly amplified and over-expressed c-*erb*B-2 gene (van de Vivjer *et al.*, 1987) was used as a positive control. Over-expression of the expected 185 kD c-*erb*B-2 immunoreactive protein correlated with amplification. In many samples, a 155 kD immunoreactive protein was also detected. Those tumours with high gene copy number expressed relatively more of the 155 kD product that the 185 kD protein. Neither the 185 kD nor the 155 kD bands were seen when the antibody 21N was pre-incubated with the immunising peptide. Figure 3 shows a typical Western blot. The tumour in track a

**Figure 3** Top panel is an immunoblot of c-*erbB*-2 protein in bladder tumours (40 μg total cellular protein per track). Lower panel shows an identical gel run in parallel and stained with Coomassie blue to assess loading.

and SKBR-3, both of which have high c-*erb*B-2 gene copy number, show high levels of expression, predominantly of the 155 kD product. A faint 155 kD band can be visualised in track k from a tumour which also contained an amplified c-*erb*B-2 gene. Comparison of the Coomassie blue stain and field biopsies from the same patient shows that the tumours in tracks d, e, n and r which have increased copy number of the c-*erb*B-2 gene also over-express the 185 kD immunoreactive product. The tumour in track g and the field biopsy in track h are from the same cystectomy specimen. Both contained amplified c-*erb*B-2 genes on Southern blotting and both showed similar levels of p185 expression. Immunoblotting was the least sensitive method of quantitation of over-expression and was therefore only applied to a limited number of samples.

The monoclonal antibody AB-3 raised against the c-terminus of c-*erb*B-2 was used to detect the c-*erb*B-2 protein product in sections of paraffin-embedded specimens from the initial tumour samples of 73 patients. 21N was used on some specimens for comparison, with similar results. Reactivity was confined to the cytoplasmic membrane of the luminal surface of mature superficial cells in normal urothelium and was very faint. All tumours which had been shown to have c-*erb*B-2 amplification showed immunoreactivity. Various distributions of the c-*erb*B-2 protein were observed. All tumour cells which reacted positively showed cytoplasmic localisation of the protein. In tumours with high levels of gene amplification, membrane and cytoplasmic reactivity was present in most tumour cells and the latter predominated (Figure 4a and c). Both cytoplasmic and membrane reactivity was abolished by pre-incubation of the antibody with the immunising peptide and no product was detected in samples where the primary antibody was omitted (Figure 4b). Those samples in which low levels of amplification had been demonstrated showed focal reactivity in discrete areas of the tumour, suggesting a heterogeneous distribution of cells which over-express c-*erb*B-2. In 40% of tumours which gene amplification, discrete clusters of cells reacted with the antibody though no over-expression of c-*erb*B-2 had been detected by other methods. These clusters of cells were often grouped on the luminal surface of the tumour. In most of these latter cases, the number of positive cells was small.

When c-*erb*B-2 immunoreactivity was compared with tumour grade, it was found that not only a large proportion of grade 3 tumours but also more than 40% of both grade 1 and grade 2 tumours showed focal reactivity (Table V). In addition seven of 20 field biopsies showed focal positive reactivity. Three of these were from bladders which contained tumours with no positive cells.

### *Patient follow-up*

Complete follow-up data for this group of patients are not yet available. However clinical information for the 2 year period of tissue collection and for 1 year afterwards has been assessed and compared with results for c-*erb*B-2 expression for a subset of 56 patients. Sixteen of 35 patients in whom

**Figure 4** Immunohistochemical localisation of c-*erbB*-2 protein in bladder tumours detected with the monoclonal antibody AB3. **a**, Membrane and cytoplasmic reactivity in most cells of a tumour which had multiple gene copies and high levels of c-*erbB*-2 mRNA; **b**, control in which primary antibody was omitted. Section from same area of the tumour shown in **a**; **c**, higher magnification of tumour shown in **a**. Both membrane and granular cytoplasmic reactivity are present. All tumour cells contain reactive material, but there is heterogeneity in the amount of product detected. Bar = 50 μm.

**Table V** Association of c-*erbB*-2 immunohistochemical reactivity and tumour grade in initial tumour samples

| | *c-*erbB*-2 immunoreactivity* | | |
|---|---|---|---|
| | + | − | *% positive tumours* |
| Grade 1 | 12 | 17 | 41 |
| Grade 2 | 11 | 7 | 61 |
| Grade 3 | 6 | 3 | 67 |

c-*erb*B-2 over-expression had been demonstrated have died of their disease or undergone radical therapy (intravesical BCG, X-irradiation or cystectomy) compared with 6/21 patients with normal expression levels. Of the remaining patients, 14/19 with c-*erb*B-2 over-expression and 5/15 with normal levels of c-*erb*B-2 expression had superficial re-occurrences at the last cystoscopic examination.

### Discussion

The need for prognostic markers for superficial bladder tumours has long been recognised. To date few potential markers have been identified, and none have good predictive value (Raghavan *et al.*, 1990). Expression of EGFR (Neal *et al.*, 1985; Berger *et al.*, 1987; Smith *et al.*, 1989) shows a significant relationship with tumour grade and stage. A few low grade superficial tumours have been reported to have increased expression of EGFR (Berger *et al.*, 1987) but the possible prognostic significance of this finding is at present unknown. Mutated *ras* genes (Fujita *et al.*, 1985; Visvanathan *et al.*, 1988) and specific deletions of the short arm of chromosome 11 (Fearon *et al.*, 1985) have been identified in a proportion of bladder tumours, though neither of these molecular markers has been shown to have prognostic significance.

The small amount of tissue available and the identification of low level gene amplification in human tumour material presents a number of technical difficulties. These have been discussed recently by Slamon *et al.* (1989) and include the need to use separate techniques to isolate each component of expression, dilution of tumour cells by normal cells, degradation of DNA, tumour cell aneuploidy, unevenness of gel loading and local variations in transfer and hybridisation. We have avoided these latter problems by the preparation of multiple blots where feasible, simultaneous hybridisation with c-*erb*B-2 and control probes, repeat hybridisations to confirm results, careful assessment of gel loading using ethidium-staining and control probes, and the use of probes on each arm of chromosome 17. These problems become less important when mRNA and protein levels of the gene of interest are studied simultaneously. We have extended the guanidine isothiocyanate method for DNA and RNA isolation to include isolation of protein from the same sample (Coombs *et al.*, 1990). This maximises the use of small samples and ensures that the DNA, RNA and protein samples compared, come from the same area of the tumour. The importance of this local sampling was exemplified clearly by our findings on the tumour from one patient, where different areas from the same large tumour showed different levels of c-*erb*B-2 gene amplification and expression. This points to the need for multiple samples with matched pathological assessment from large tumours. Our finding that intact DNA can be isolated from exfoliated cells collected from urine may provide the basis for a non-invasive assay in the future.

We have shown that amplification and over-expression of c-*erb*B-2 in transitional cell tumours, as in a number of epithelial tumour types, is a frequent event. Twelve out of 86 (14%) had an amplified gene at the time of first biospy. This frequency is in the range (11–30%) reported for breast and ovarian tumours (Zhou *et al.*, 1989; Slamon *et al.*, 1987, 1989). Amplification was more frequent and gene copy number higher in high grade tumours. The correlation found between tumour grade and gene amplification suggests that amplification is linked directly or indirectly to disease progression. The absence of gene amplification in the control samples and field biopsies shows that amplification of c-*erb*B-2 is specific to overt transitional cell carcinoma in the urinary bladder. In the only field biopsy in which gene amplification was detected, it was found that most of the bladder mucosa in this cystectomy specimen was replaced by tumour.

The patients from whom additional biopsies were obtained represented a random group of patients. In this group, 7/23 patients developed amplification during the course of the study and although this was not associated with increased tumour grade, it appeared to be associated with disease progression. We have avoided the term 'recurrences' for those tumours that developed subsequent to the initial resection, since in many patients they occur at different sites in the bladder and often develop within an unstable urothelium which shows widespread field changes. Thus, in many cases such tumours are likely to represent new 'occurrences'. It is clear that future studies concerning the progression of neoplastic disease in the bladder must address this question in detail.

If amplification of a gene is relevant to the pathogenesis of the disease, changes in gene expression must be expected. Some amplified genes are not expressed in the relevant tumours, e.g. c-*erb*A, which in breast tumours is often co-amplified with c-*erb*B-2 (Tavassoli *et al.*, 1989). Although our assessment of levels of c-*erbB*-2 transcript were hampered by the variable levels of RNA degradation, it was possible to demonstrate that levels of c-*erb*B-2 transcript parallelled amplification and that some high grade or clinically aggressive tumours had raised levels of transcript in the absence of gene amplification. In one case, raised levels of mRNA preceded the detection of gene amplification in a subsequent biopsy.

Immunoblotting revealed two immunoreactive proteins at 185 kD and approximately 155 kD. De Potter *et al.* (1989) have suggested that these may represent the c-*erbB*-2 cell membrane protein and a 155 kD protein associated with the mitochondrial membrane. The smaller of these may represent a partially glycosylated form of the protein since inhibition of N-linked glycosylation by tunicamycin has been shown to produce an immunoprecipitable protein of 155–160 kD in human cells which express c-*erb*B-2 (Akiyama *et al.*, 1986). Immunoblotting was the least sensitive assay of c-*erb*B-2 expression. Only large differences could be demonstrated clearly. This finding is in keeping with those of Slamon *et al.* (1989) in breast tumours and may reflect dilution of tumour cell protein by variable amounts of protein from other cells, stromal elements and necrotic debris.

Immunocytochemistry showed that the c-*erb*B-2 gene product is confined to the luminal membrane of mature surface cells in normal urothelium. In bladder tumours, we have found both cytoplasmic and membrane reactivity. In contrast to reports on breast tumours, most c-*erb*B-2 protein in these transitional cell tumours was cytoplasmic, and this was particularly pronounced in tumours with high gene copy number and mRNA expression. In this context, it may be significant that high levels of the 155 kD protein were detected in such tumours. Good correlation was found between the number of positive cells, the degree of gene amplification and amount of mRNA detected. Granular cytoplasmic reactivity, though less prominant that membrane reactivity, has been demonstrated in breast tumours using antibodies to different epitopes on the protein (both internal and external) (Berger *et al.*, 1988; Gusterson *et al.*, 1988; van de Vijver *et al.*, 1988) and this correlates with c-*erb*B-2 gene amplification and membrane reactivity (Gusterson *et al.*, 1988).

In colon carcinomas, c-*erb*B-2 protein appears to be primarily cytoplasmic and correlates with gene amplification (D'Emilia *et al.*, 1989). The close correlation of amplificaiton of c-*erb*B-2 and cytoplasmic immunoreactivity in the present and other studies argues that the cytoplasmic product does represent a form of the c-*erb*B-2 protein. It will be important to examine these two protein forms and cellular location more fully.

The significance of the heterogeneous distribution of over-expression of the c-*erb*B-2 protein is not clear. Possible explanations include technical artefacts, local conditions within the tumour which may induce or inhibit c-*erb*B-2 expression and the possibility that some tumours represent mosaics of cells with different molecular lesions. McCann *et al.* (1990) in a study of 48 bladder tumours, reported c-*erb*B-2 staining in only one tumour. The reason for this discrepancy is not clear but may reflect differences in scoring thresholds and/or criteria. Wright *et al.* (1990) have recently reported that in a series of 44 bladder tumours, 36% showed c-*erb*B-2 immunocytochemical staining on frozen tissues sections. Our findings on paraffin-embedded material show similar frequencies. These latter authors reported a significant reduction in staining sensitivity on paraffin-embedded material which may suggest that the apparent differences between our findings and those of McCann *et al.* (1990) are indeed attributable to differences in scoring thresholds.

Forty percent of tumours with no detectable c-*erb*B-2 amplification or over-expression which could be detected by Northern or Western analysis showed immunoreactivity. Similar findings have been reported in breast tumours with no gene amplification (Slamon *et al.*, 1989; Berger *et al.*, 1988). This suggests that c-*erb*B-2 may be implicated in the development of a higher proportion of breast and bladder tumours than is suggested by the identification of gene amplification alone. Thus, immunocytochemistry may be the most sensitive assay for future studies.

The function of the c-*erb*B-2 gene product in normal and transformed epithelial cells remains unknown, though its frequent over-expression in only certain tumours and high level expression in only certain normal cell types e.g. colon (Cohen *et al.*, 1989) indicates a likely tissue specific role. Binding of EGF to its receptor leads to an increase in the phosphorylation and tyrosine kinase activity of c-*erb*B-2 (Akiyama *et al.*, 1988). It has also been shown that over-expression of EGFR and c-*neu* in NIH3T3 cells is sufficient for transformation (Kokai *et al.*, 1989). This interaction suggests that in the bladder, where surface cells are bathed in EGF, over-expression of either or both of these receptor proteins might lead to a proliferative advantage. In this case, therapies based on receptor targeting may be particularly useful.

Since 25% of transitional cell tumours progress clinically during the first 3 years after presentation and only 7% during the subsequent 7 years (Pryor, 1973), it should be possible to assess the prognostic significance of c-*erb*B-2 expression within a relatively short period of time. It was notable that amplification was detected in a superficial grade 1 tumour and over-expression in a relatively large subset of grade 1 tumours. Follow-up of these patients will be of particular interest. Since inaccuracies in the assessment of grade and stage prevent their use as indices of progression, evaluation of c-*erb*B-2 as a prognostic marker will depend on complete clinical follow-up of a large series of patients.

Our preliminary results, based on clinical data available for the period of 2 years during which tissues were collected and for 1 year following collection of the last samples indicate that c-*erb*B-2 over-expression may be associated with the incidence of tumour re-occurrences and with advancing disease. The finding that c-*erb*B-2 over-expression appears to precede disease progression suggests that this may represent a true prognostic indicator in transitional cell carcinoma.

We are extremely grateful to the Consultant surgeons and their staff who co-operated in this study. We also thank Dr G. Currie for valuable discussions, encouragement and advice and Mrs J. Marr for careful preparation of the manuscript.

### References

ABEL, P.D., HENDERSON, D., BENNETT, M.K., HALL, R.R. & WILLIAMS, G. (1988). Differing interpretations by pathologists of the pT category and grade of transitional cell cancer of the bladder. *Br. J. Urol.*, **62**, 339.

AKIYAMA, T., SUDO, C., OGAWARA, H., TOYOSHIMA, K. & YAMAMOTO, T. (1986). The product of the human c-erbB-2 gene: a 185 kilodalton glycoprotein with tyrosine kinase activity. *Science*, **232**, 1644.

AKIYAMA, T., SAITO, T., OGAWARA, H., TOYOSHIMA, K. & YAMAMOTO, T. (1988). Tumour promoter and epidermal growth factor stimulate phosphorylation of the c-erbB-2 gene product in MKN-7 human adenocarcinoma cells. *Mol. & Cell. Biol.*, **8**, 1019.

BARGMANN, C.I., HUNG, M.-C. & WEINBERG, R.A. (1986*a*). The neu oncogene encodes an epidermal growth factor receptor-related protein. *Nature*, **319**, 226.

BARGMANN, C.I., HUNG, M.-C., & WEINBERG, R.A. (1986*b*) Multiple independent activations of the neu oncogene by a point mutation altering the transmembrane domain of p185. *Cell*, **45**, 649.

BENCHIMOL, S., LAMB, P., CRAWFORD, L.V. & 4 others (1985). Transformation associated p53 protein is encoded by a gene on human chromosome 17. *Somatic Cell Mol. Genet.*, **11**, 505.

BERGER, M.S., GREENFIELD, C., GULLICK, W.J. & 5 others (1987). Evaluation of epidermal growth factor receptors in bladder tumours. *Br. J. Cancer*, **56**, 533.

BERGER, M.S., LOCHER, G.W., SAURER, S. & 4 others (1988). Correlation of c-erbB-2 gene amplification and protein expression in human breast carcinoma with nodal status and nuclear grading. *Cancer Res.*, **48**, 1238.

BRADFORD, M. (1976). A rapid and sensitive method for the quantitation of microgram quantities of protein utilising the principle of protein-dye binding. *Analytical Biochem.*, **72**, 248.

COHEN, J.A., WEINER, D.B., MORE, K.F. & 5 others (1989). Expression pattern of the neu (NGL) gene-encoded growth factor receptor protein ($p185^{neu}$) in normal and transformed epithelial tissues of the digestive tract. *Oncogene*, **4**, 81.

COOMBS, L.M., KNOWLES, M.A. & MILROY, E. (1989). Her2 (c-erbB-2, neu, Mac117) amplification and expression in transitional cell carcinoma. *Urological Res.*, **17**, 345.

COOMBS, L.M., PIGOTT, D., PROCTOR, A., EYDMANN, M., DENNER, J. & KNOWLES, M.A. (1990). Simultaneous isolation of DNA, RNA and antigenic protein exhibiting kinase activity from small tumour samples using guanidine isothiocyanate. *Analytical Biochem.*, **188**, 338.

COUSSENS, L., YANG-FENG, T.L., LIAO, Y.-C. & 9 others (1985). Tryosine kinase receptor with extensive homology to EGF receptor shares chromosomal location with neu oncogene. *Science*, **230**, 1132.

D'EMILIA, J., BULOVAS, K., D'ERCOLE, K., WOLF, B., STEELE, G. Jr. & SUMMERHAYES, I.C. (1989). Expression of the c-erbB-2 gene product (p185) at different stages of neoplastic progression in the colon. *Oncogene*, **4**, 1233.

DE POTTER, C.R., QUATACKER, J., MAERTENS, G. & 5 others (1989). The subcellular localisation of the neu protein in human normal and neoplastic cells. *Int. J. Cancer*, **44**, 969.

FARBER, E. (1984). The multistep nature of cancer development. *Cancer Res.*, **44**, 4217.

FEARON, E.R., FEINBERG, A.P., HAMILTON, S.H. & VOGELSTEIN, B. (1985). Loss of genes on the short arm of chromosome 11 in bladder cancer. *Nature*, **318**, 377.

FEINBERG, A.P. & VOGELSTEIN, B. (1983). A technique for radiolabeling DNA restriction endonuclease fragments to high specific activity. *Analytical Biochem.*, **132**, 6.

FORT, Ph., MARTY, L., PIECHACZYK, M. & 4 others (1985). Various rat adult tissues express only one major mRNA species from the glyceraldehyde 3-phosphate-dehydrogenase multigenic family. *Neucleic Acids Res.*, **13**, 1431.

FOULDS, L. (1975). *Neoplastic Development*, 1 & 2. Academic Press: London.

FUJITA, J., SRIVASTAVA, S.K., KRAUS, M.H., RHIM, J.S., TRONICK, S.R. & AARONSON, S.A. (1985). Frequency of molecular alterations affecting ras proto-oncogenes in human urinary tract tumours. *Proc. Natl Acad. Sci. USA*, **82**, 3849.

GULLICK, W.J., BERGER, M.S., BENNETT, P.L.P., ROTHBARD, J.B. & WATERFIELD, M.D. (1987). Expression of the c-erbB-2 protein in normal and transformed cells. *Int. J. Cancer*, **40**, 246.

GUSTERSON, B.A., GULLICK, W.J., VENTER, D.J. & 5 others (1988). Immunohistochemical localisation of c-erbB-2 in human breast carcinomas. *Molecular & Cellular Probes*, **2**, 383.

HSU, S.M., RAINE, L. & FANGER, H. (1981). Use of avidin-biotin-peroxidase complex (ABC) in immunoperoxidase techniques: a comparison between ABC and unlabelled antibody (PAP) procedures. *J. Histochem. Cytochem.*, **29**, 577.

KING, C.R., KRAUS, M.H. & AARONSON, S.A. (1985). Amplification of a novel v-erbB related gene in a human mammary carcinoma. *Science*, **229**, 974.

KOKAI, Y., MYERS, J.N., WADA, T. & 5 others (1989). Synergistic interaction of p185c-neu and the EGF receptor leads to transformation of rodent fibroblasts. *Cell*, **58**, 287.

LAEMMLI, U.K. (1970). Cleavage of structural proteins during the assembly of the head of Bacteriophage T4. *Nature*, **227**, 680.

LAU, Y.F. & KAN, Y.W. (1984). Direct isolation of the functional human thymidine kinase gene with a cosmid shuttle vector. *Proc. Natl Acad. Sci. USA*, **81**, 414.

LEMOINE, N.R., STADDON, S., DICKSON, C., BARNES, D.M. & GULLICK, W.J. (1990). Absence of activating transmembrane mutations in the c-erbB-2 proto-oncogene in human breast cancer. *Oncogene*, **5**, 237.

McCANN, A., DERVAN, P.A., JOHNSTON, P.A., GULLICK, W.J. & CARNEY, D.N. (1990). c-erbB-2 oncoprotein expression in primary human tumours. *Cancer*, **65**, 88.

NEAL, D.E., MARSH, C., BENNETT, M.K. & 4 others (1985). Epidermal growth factor receptors in human bladder cancer: comparison of invasive and superficial tumours. *Lancet*, **i**, 366,

OFFICE OF POPULATION CONSENSUSES AND STUDIES, M.B.1 *1971–1984. Cancer Statistics.* HMSO.

OFFICE OF POPULATION CONSENSUSES AND STUDIES, D.H.1 *1969–1987. Mortality Statistics.* HMSO.

OOMS, E.C.M., ANDERSON, W.A.D., ALONS, C.L., BOON, M.E. & VELHUIZEN, R.W. (1983). Analysis of the performance of pathologists in the grading of bladder tumours. *Human Pathology*, **14**, 140.

PRYOR, J.P. (1973). Factors influencing the survival of patients with transitional cell tumours of the urinary bladder. *Br. J. Urol.*, **45**, 586.

RAGHAVAN, D., SHIPLEY, W.U., GARNICK, M.B., RUSSELL, P.J. & RICHIE, J.P. (1990). Biology and management of bladder cancer. *N Eng. J. Med.*, **322**, 1129.

SCHECHTER, A.L., STERN, D.F., VAIDYANATHAN, L. & 4 others (1984). The neu oncogene: an erb-B-related gene encoding a 185,000-Mr tumour antigen. *Nature*, **312**, 513.

SCHECHTER, A.L., HUNG, M.-C., VAIDYANATHAN, L. & 5 others (1985). The neu gene: an erbB-homologous gene distinct from and unlinked to the gene encoding the EGF receptor. *Science*, **229**, 976.

SEMBA, K., KAMATA, N., TOYOSHIMA, K. & YAMAMOTO, T. (1985). A v-erbB-related protooncogene, c-erbB-2, is distinct from the c-erbB-1/epidermal growth factor-receptor gene and is amplified in a human salivary gland adenocarcinoma. *Proc. Natl Acad. Sci. USA*, **82**, 6497.

SHIH, C., PADHY, L.C., MURRAY, M. & WEINBERG, R.A. (1981). Transforming genes of carcinomas and neuroblastomas introduced into mouse fibroblasts. *Nature*, **290**, 261.

SLAMON, D.J., CLARK, G.M., WONG, S.G., LEVIN, W.J., ULLRICH, A. & McGUIRE, W.L. (1987). Human breast cancer: correlation of relapse and survival with amplification of the HER-2/neu oncogene. *Science*, **235**, 117.

SLAMON, D.J., GODOLPHIN, W., JONES, L.A. & 8 others (1989). Studies of the Her2/neu proto-oncogene in human breast and ovarian cancer. *Science*, **244**, 707.

SMITH, K., FENNELLY, J.A., NEAL, D.E., HALL, R.R. & HARRIS, A.L. (1989). Characterization and quantitation of the epidermal growth factor receptor in invasive and superficial bladder tumours. *Cancer Res.*, **49**, 5810.

SOUTHERN, E.M. (1975). Detection of specific sequences among DNA fragments separated by gel electrophoresis. *J. Mol. Biol.*, **98**, 503.

TAVASSOLI, M., QUIRKE, P., FARZANEH, F., LOCK, N.J., MAYNE, L.V. & KIRKHAM, N. (1989). c-erbB-2/c-erbA co-amplification indicative of lymph node metastasis, and c-myc amplification of high tumour grade in human breast carcinoma. *Br. J. Cancer*, **60**, 505.

THOMAS, P. (1980). Hybridization of denatured RNA and small DNA fragments transferred to nitrocellulose. *Proc. Natl Acad. Sci. USA*, **77**, 5201.

TOWBIN, H., STAEHELIN, T. & GORDON, J. (1979). Electrophoretic transfer of proteins from polyacrylamide gels to nitrocellulose sheets: procedure and some applications. *Proc. Natl Acad. Sci. USA*, **76**, 4350.

VAN DE VIJVER, M., VAN DE BERSSELAAR, R., DEVILEE, P., CORNELISSE, C., PETERSE, J. & NUSSE, R. (1987). Amplification of the neu (c-erbB-2) oncogene in human mammary tumors is relatively frequent and is often accompanied by amplification of the linked c-erbA oncogene. *Mol. & Cell. Biol.*, **7**, 2019.

VAN DE VIJVER, M.J., PETERSE, J.L., MOOI, W.J. & 4 others (1988). Neu-protein overexpression in breast cancer: association with comedo-type ductal carcinoma in situ and limited prognostic value in stage II breast cancer. *New Eng J. Med.*, **319**, 1239.

VAN TUINEN, P., RICH, D.C., SUMMERS, K.M. & LEDBETTER, D.H. (1987). Regional mapping panel for human chromosome 17: application to neurofibromatosis type I. *Genomics*, **1**, 374.

VISVANATHAN, K.V., POCOCK, R.D. & SUMMERHAYES, I.C. (1988). Preferential and novel activation of H-ras in human bladder carcinomas. *Oncogene Res.*, **3**, 77.

WRIGHT, C., MELLON, D.E., NEAL, D.E., JOHNSON, P., CORBETT, I.P. & HORNE, C.H.W. (1990). Expression of c-erbB-2 protein product in bladder cancer. *Br. J. Cancer*, **62**, 764.

YAMAMOTO, T., IKAWA, S., AKIYAMA, T. & 5 others (1986). Similarity of protein encoded by the human c-erbB-2 gene to epidermal growth factor receptor. *Nature*, **319**, 230.

YOKOTA, J., YAMAMOTO, T., TOYOSHIMA, K. & 4 others (1986). Amplification of c-erbB-2 oncogene in human adenocarcinomas in vivo. *Lancet*, **i**, 765.

ZHOU, D.-J., AHUJA, H. & CLINE, M.J. (1989). Proto-oncogene abnormalities in human breast cancer: c-ERBB-2 amplification does not correlate with recurrence of disease. *Oncogene*, **4**, 105.